# Kinetics of Austenite Decomposition in 54SiCr6 Steel during Continuous Slow Cooling Conditions

**DOI:** 10.3390/ma16134619

**Published:** 2023-06-27

**Authors:** Aleksandr Gokhman, Petr Motyčka, Pavel Salvetr, Zbyšek Nový, Jakub Kotous, Arkadii Briukhanov, Ján Džugan

**Affiliations:** 1 COMTES FHT a.s., Prumyslova 995, 334 41 Dobrany, Czech Republic; alexander.gokhman@gmail.com (A.G.); petr.motycka@comtesfht.cz (P.M.);; 2 Department of Physics, South Ukrainian National Pedagogical University (SUNPU), Staroprotfrankivska 26, 65020 Odessa, Ukraine

**Keywords:** 54SiCr6 steel, dilatometry, metallography, transformation kinetics, local activation energy, local Avrami exponent

## Abstract

In this study, dilatometry and metallography were used to investigate the effect of silicon and copper alloying on the decomposition kinetics of 54SiCr6 steel during continuous slow cooling. It is different from the published literature for using the approach of the local activation energy of the austenite decomposition *E_f_* and the local Avrami exponent *n* of the volume fraction of the transformed phase *f* to study the kinetics of austenite-pearlitic transformation in cooled 54SiCr steel at slow cooling rates. The Johnson–Mehl–Avrami equation was used to determine the dependence of the local activation energy for austenite decomposition *E_f_* and the local Avrami exponent *n* on the volume fraction of the transformed phase *f*. The mechanism of the austenite decomposition was analysed based on the calculated values of *n*. Both the local and average activation energies were used to evaluate the alloying effect, and the results were compared with those obtained from other methods. The type of microstructure formed as a result of cooling at rates of 0.5 K/s, 0.3 K/s, 0.1 K/s and 0.05 K/s was determined. The effects of changes in the cooling rate and the content of silicon (1.5–2.5 wt.%) and copper (0.12–1.47 wt.%) on the dimension of nucleation and growth kinetics of the transformed phase were studied. It was revealed that the pearlite microstructure was formed predominantly in 54SiCr6 steel as a result of continuous cooling at slow cooling rates. It was also found that alloying this steel with copper led to a significant decrease in the value of *E_f_*_,_ as well as to a change in the mechanism of the kinetics of the austenite-pearlite transformation, which was realised in predominantly two- and three-dimensional nucleation and growth at a constant nucleation rate. At the same time, alloying this steel with silicon led only to a slight change in *E_f_*. The results of the study of 54SiCr steel presented the dependence of the activation energy of transformation and the local Avrami exponent on the volume fraction of the transformed phase at a given cooling rate at different copper and silicon contents. In addition, the study provides insight into the mechanism of kinetics in cooled 54SiCr steel as a function of the cooling rate.

## 1. Introduction

Low-alloyed medium-carbon spring steels have excellent mechanical properties, making them suitable for reducing the weight of road and rail vehicle springs. Steels are usually alloyed with other elements—such as chromium, silicon, manganese, vanadium, molybdenum and nickel—to improve their properties [1,2,3]. As for transport technology production in general, further enhancement of the strength, toughness and fatigue parameters is required to improve fuel consumption. Cost effectiveness is also demanded [4], which is where new, advanced modifications of heat treatments and thermomechanical treatments of cheaper, low-alloyed steels come in. Advanced processing also leads to microstructure refinement, which is connected to acquiring a considerably better combination of properties [5]. For the accurate prediction and control of their microstructure during heat or thermomechanical treatments, information about the various transformation products arising during continuous cooling is needed. A thorough understanding of the phase transformation kinetics is also essential for controlling the steel products’ microstructure.

Ferrite, cementite and pearlite are the principal constituents of carbon steels’ microstructure when subjected to slow cooling, as they prevent the formation of a metastable phase in the form of martensite and/or bainite. The proportions of these constituents during slow cooling are determined by the carbon content of the steel.

The kinetics of phase transformation in eutectoid carbon steel 0.795 C, 0.91 Mn, 0.49 Si, 0.029 S, 0.018 P, 0.084 Al, 0.049 Cu, 0.062 Cr, 0.003 Sn, 0.014 Ni, 0.002 Mo wt.% was studied in [6], and only an austenite-to-pearlite transformation was found. A metallographic examination of partially transformed samples, obtained via isothermal treatment at 690 and 640 °C and quenching in water, revealed that pearlite nucleates predominantly at grain edges and possibly corners, which was also established via a theoretical analysis of the kinetics of austenite decomposition (the Avrami exponent *n* was found to be between 1.9 and 2.7). However, the good agreement between the calculated and measured transformation kinetics for the slowest cooling rate runs contrary to the site saturation condition suggested by Cahn.

Carbon steel 1025 0.25 C, 0.46 Mn, 0.21 Si wt.% was studied in [7]. In accordance with the effect of carbon on the decomposition of austenite, the occurrence of both austenite-to-ferrite and austenite-to-pearlite transformations was found in this study.

In [8], a model of the kinetics of a continuous phase transformation was applied to evaluate the austenitisation of 55CrMo steel 0.58 C, 0.24 Si, 0.89 Mn, 0.22 Mo, 0.89 Cr, 0.017 Ni, 0.004 V, 0.015 P, 0.002 S wt.%. Based on the dilatometric measurement for heating at the rates of 0.05 K/s, 0.3 K/s, 1.0 K/s, 3.0 K/s, 5.0 K/s, 10.0 K/s, 20 K/s, 30 K/s and 50 K/s, the activation energy for the transformation *E_f_* was found to be 726,400 J/mol, and the Avrami exponent *n* was 1.23 for this steel. To do this, a regression analysis was carried out in [8] to estimate the parameters in the non-isothermal Johnson–Mehl–Avrami (JMA) equation. This equation is strictly valid when conditions such as the following are not violated: pure site saturation at time *t* = 0, pure continuous nucleation, large undercooling or overheating to form a high driving force, or random isotropic nucleation [9]. The role of the boundary conditions was also discussed in [9]. The comparison of the calculation results and the experiment results in [8] shows that the simulation results are consistent with the experiment results. In other words, the JMA equation can describe the austenitisation of 55CrMo steel well. According to [10], the value of *n* means that in the steel under study in [8], one-dimensional nucleation occurs and a new phase grows at a nucleation rate close to zero. A large value of *E_f_* in comparison with the activation energy of carbon diffusion through the grain boundary, as well as in the volume in austenite, is consistent with the chemical composition of the steel [8].

The purpose of the paper was the application of the JMA equation to study the kinetics of phase transformation during the cooling of different grades of 54SiCr6 (BX, B and D steels in Table 1) with a carbon content of 0.57 wt.% at the slow cooling rates of 0.05 K/s, 0.1 K/s, 0.3 K/s and 0.5 K/s. The similarity of the chemical composition of 55CrMo steel and the studied steels made it possible to use the JMA equation to process the dilatometry data. In contrast to the study [8], which used dilatometry data related only to three values of the transformed volume fraction *f*, the presented studies use dilatometry data for all values of *f*. The local activation energy for the phase transition, *E_f_*, and the local Avrami exponent, *n*, were selected as kinetics parameters according to the approaches in [8,10,11,12,13]. Steel 54SiCr6 was chosen as a part of broader research on the influence of Si, Cr and Cu on the microstructure and mechanical properties of steel 54SiCr6 and the correlation between them in middle-carbon spring steels [14,15,16,17,18,19]. The carbon content in this steel is almost the same as in the steel studied in [8], while the Si content (1.5 and 2.5 wt.%) exceeds that in the steel in [6] (0.24 wt.%).

## 2. Materials and Methods

The chemical compositions of different grades of medium carbon spring steel 54SiCr6 (steels BX, B and D) are given in Table 1. They were determined using a Q4 Tasman optical emission spectrometer (Bruker Elemental GmbH, Kalkar, Germany). The steels were melted in a vacuum induction furnace and cast in 45-kg ingots.

The ingots were heated to 1050 °C, hot rolled into sheets with a thickness of 14 mm and air cooled. Normalisation annealing was carried out at 850 °C for 40 min to obtain a uniform microstructure with a refined grain size.

To investigate the effects of Si, the Si content was varied in the range of 1.5–2.5 wt.% of silicon in 54SiCr6 steel (steels B and D). The effect of Cu was studied and compared to Cu-free (0.12 wt.%) and Cu-alloyed samples with 1.47 wt.% of Cu (steels BX and B).

Metallographic samples were prepared through grinding and polishing, and the microstructure was revealed through etching with a 2% Nital reagent. The samples were examined using a Zeiss Observer Z1m optical microscope (Zeiss, Oberkochen, Germany). The dilatometric samples were prepared through grinding and polishing, and the microstructure was revealed with a 3% Nital reagent. A L75PT dilatometer (Linseis, Selb, Germany) was used to observe the microstructure after austenite decomposition at cooling after 15 min of austenitisation at 900 °C. The same austenitisation temperature was used in previous works [19,20]. The dilatometric samples were cylindrical with a diameter of 4 mm and a length of 10 mm. Experiments were performed in pure nitrogen. Specimens were held between quartz glass push-rods with a contact force of 0.3 N. To avoid martensitic transformation, continuous slow cooling was performed at cooling rates β = 0.05 K/s, 0.1 K/s, 0.3 K/s and 0.5 K/s. Slow cooling was broken after specified times through rapid cooling to achieve the visualisation of the different stages of pearlitic transformation. The conditions of pearlitic transformation for individual stages are listed in Table 2. 

## 3. Results

### 3.1. Results of Metallographic Examinations

**Figure 1:**
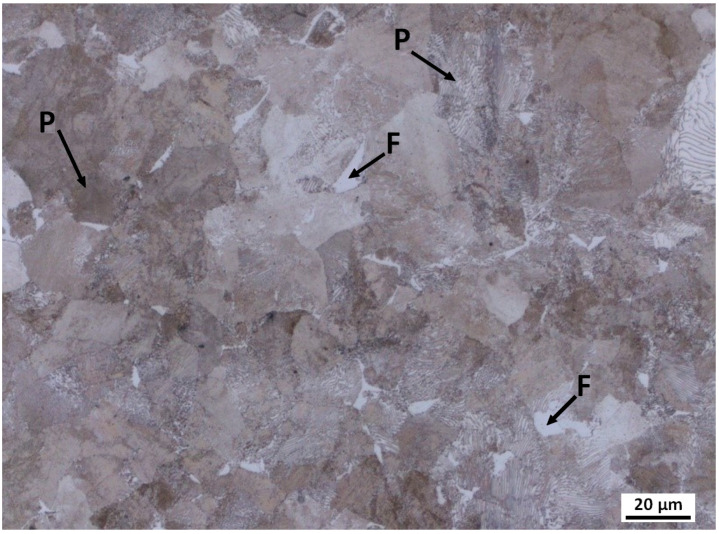
Optical metallography of the microstructure of steel BX after the completion of continuous cooling at a cooling rate of 0.05 K/s (F = Ferrite, P = Pearlite).

The pearlite phase predominated in steels BX, B and D after the completion of continuous cooling at all given cooling rates. Figure 1 shows an example of the results of optical metallography of the microstructure of steel BX continuously cooled at a cooling rate of 0.05 K/s.

Additionally, to demonstrate the kinetics of the phase transformation, the samples were examined after partially completed continuous cooling followed by quenching with water at a rate of 100 K/s. The results for steels B and D were very similar, while for steel BX there was a difference in microstructure evolution compared to steels B and D. 

**Figure 2:**
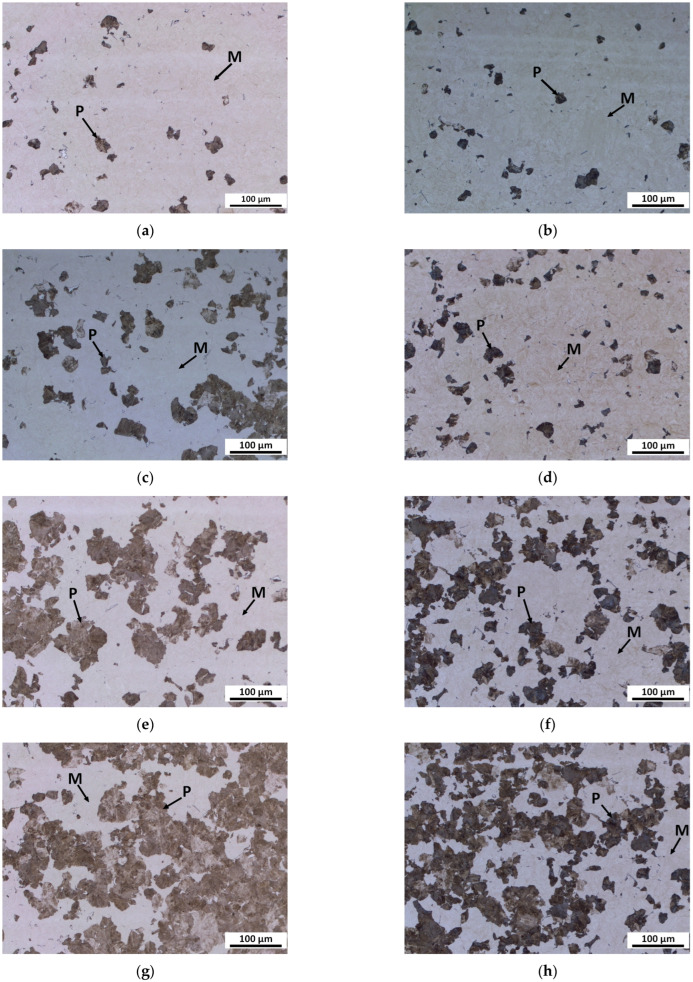
Optical metallography of the microstructure of steels BX and B continuously cooled at a cooling rate of 0.3 K/s to different stages of pearlite formation: initial stage of pearlite formation of steel BX (**a**) and steel B (**b**); advanced stage of pearlite formation of steel BX (**c**) and steel B (**d**); more advanced stage of pearlite formation of steel BX (**e**) and steel B (**f**); later stage of pearlite formation of steel BX (**g**) and steel B (**h**); P = pearlite, M = martensite.

Figure 2 shows an example of the results of optical metallography of the microstructure of steels BX and B continuously cooled at a cooling rate of 0.3 K/s to four stages of pearlite formation.

At the initial stage, pearlite was formed from both steels’ previous austenite grains (PAG). However, the predominant formation of pearlite particles in the triple junction and edges of the PAG in steel B was different from that of steel BX, where pearlite particles were observed both on and inside the PAG.

At the advanced stage, the pearlite particles at the PAG grew faster than inside the PAG in both steels BX and B, while the difference in the spatial distribution of the pearlite particles was preserved.

At the more advanced stage, larger pearlite particles were visible mainly at the PAG boundaries but also inside the PAG. There were still very small pearlite particles visible in the BX steel, which were not common in the B steel. The pearlite almost completely enveloped the PAG, and some smaller pearlite islands were visible in the interior of the PAG in steel B. However, large pearlite islands were regularly displaced inside the PAG and to the boundary in the BX steel.

At the later stage, the coarse pearlite particles were visible at the PAG boundary as well as in the interior, and the microstructure features of BX steel were very similar to B steel. However, in addition to rough pearlite islands, many very fine particles were also still visible in BX steel.

### 3.2. Results of Dilatometry Studies

**Figure 3:**
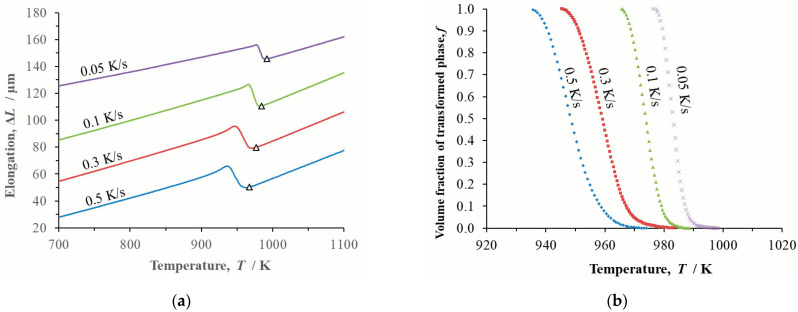
Dilatometric study of steel BX continuously cooled at cooling rates *β* = 0.05 K/s, 0.1 K/s, 0.3 K/s and 0.5 K/s: (**a**) temperature dependence of changes to the length of the dilatometric sample, Δ*L* triangles mark the onset of phase transformation; (**b**) temperature dependence of the transformed volume fraction *f*.

**Figure 4:**
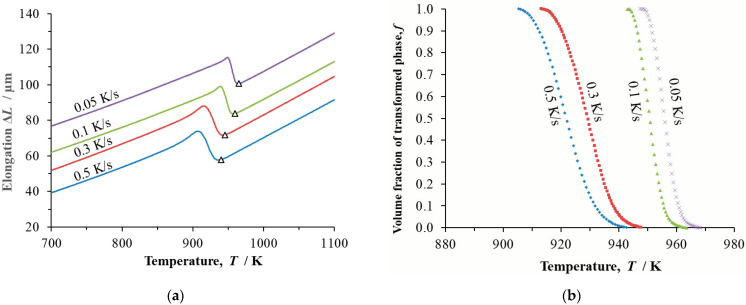
Dilatometric study of steel B continuously cooled at cooling rates *β* = 0.05 K/s, 0.1 K/s, 0.3 K/s and 0.5 K/s: (**a**) temperature dependence of changes to the length of the dilatometric sample, Δ*L*; triangles mark the onset of phase transformation; (**b**) temperature dependence of the transformed volume fraction *f*.

**Figure 5:**
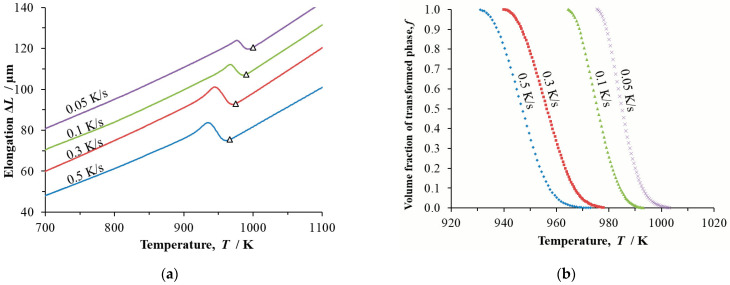
Dilatometric study of steel D continuously cooled at cooling rates *β* = 0.05 K/s, 0.1 K/s, 0.3 K/s and 0.5 K/s: (**a**) temperature dependence of changes to the length of the dilatometric sample, Δ*L*; triangles mark the onset of phase transformation; (**b**) temperature dependence of the transformed volume fraction *f*.

Dilatometry records of continuous cooling in the form of temperature and transformed volume fraction were determined for the steels at cooling rates *β* = 0.05 K/s, 0.1 K/s, 0.3 K/s and 0.5 K/s (Figure 3, Figure 4 and Figure 5).

The Johnson–Mehl–Avrami–Kolmogorov equation was used to analyse the kinetics of austenite decomposition:

(1) $$f=1-exp\left(-{\left|\frac{K\left(T-T_{0}\right)}{\beta }\right|}^{n}\right)$$

where *T*_0_ is an onset of temperature of the phase transition and *K* is a temperature-dependent kinetic parameter related to the growth rate and nucleation frequency, which was expressed as

(2) $$K=K_{0}\exp\left(-\frac{E_{f}}{RT}\right)$$

where *K*_0_ is a frequency factor (constant), *E_f_* is the local activation energy of the phase transition and *R* is a gas constant.

*Ef* as a function on the volume fraction of transformed phase *f* was calculated using Equation (3) from [8,10,11,12,13]:

(3) $$ln\left(\frac{T_{f}^{2}}{\beta }\right)=\frac{E_{f}}{RT_{f}}+C$$

where *T_f_* is the temperature corresponding to the volume fraction of transformed phase *f*, and *C* is a constant, which is determined in [21] as $ln\frac{E_{f}}{RK_{0}}+ln\beta_{f}$, where $\beta_{f}$ is a state variable.

The ratio of the local activation energy *E_f_* to *R* was regarded as the slope of the straight line between $ln\left(\frac{T_{f}^{2}}{\beta }\right)$ and $\frac{1}{T_{f}}$ for the set of cooling rates *β* used.

**Figure 6:**
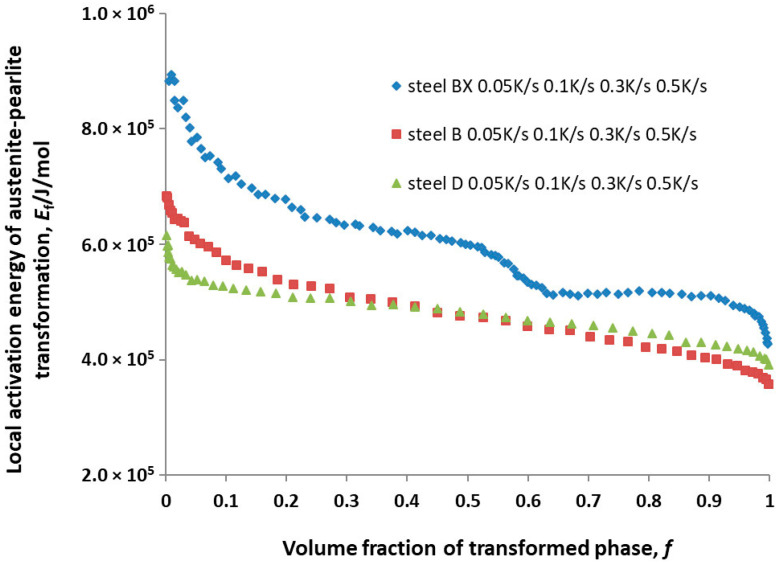
Local activation energy for the austenite-to-pearlite transformation *E_f_* as a function on the volume fraction of transformed phase *f* calculated in [8,10,11,12,13].

The calculation results for *E_f_* for steels BX, B and D are shown in Figure 6.

The mechanism of the austenite-to-pearlite transformation was analysed based on the value of the local Avrami exponent *n* calculated using Equation (4) from [8,10,11,12,13]:

(4) $$n=-\frac{R\partial ln\left[-ln\left(1-f\right)\right]}{E_{f}\partial \left(\frac{1}{T_{f}}\right)}$$

According to [11,22], the value of *n* in the range (3–4) corresponds to the three-dimensional nucleation and growth of a new phase at a constant nucleation rate. In the range (2–3), *n* corresponds to the two-dimensional nucleation and growth of a new phase at a constant nucleation rate. In the range (1–2), *n* corresponds to a one-dimensional nucleation and growth of a new phase at a near-zero nucleation rate. Values of *n* greater than 4 are not taken into consideration, since they have no physical significance [11].

**Figure 7:**
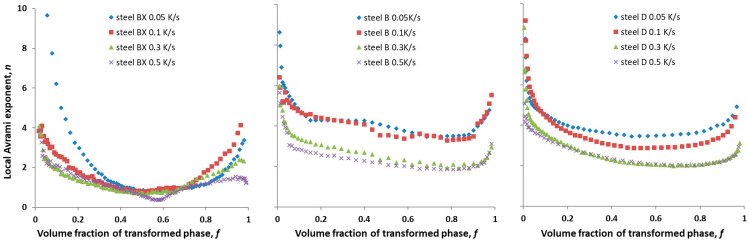
The Local Avrami exponent *n* for low cooling rates as a function of volume fraction of transformed phase *f* for steels BX, B and D.

Dependences *n*(*f*) for steels BX, B and D cooled at different cooling rates are shown in Figure 7.

For steel BX, at the cooling rates of 0.5 K/s, 0.3 K/s and 0.1 K/s, the local Avrami exponent *n* varied in the range of (2–4) at *f* < 0.20 and in the range (2–1) at *f* > 0.20. Therefore, the austenite-to-pearlite transformation process can be divided into two stages at the given cooling rates. 

Two- and three-dimensional nucleation and growth were at a constant nucleation rate at *f* < 0.20, whereas one-dimensional nucleation and growth were at a near-zero nucleation rate at *f* > 0.20. 

Similarly, at a cooling rate of 0.05 K/s, *n* varied in the range of (2–4) at 0.14 < *f* < 0.33 and in the range (1–2) at 0.33 < *f* < 0.95. This result indicated that two- and three-dimensional nucleation and growth at a constant nucleation rate at 0.14 < *f* < 0.33 and one-dimensional nucleation and growth at a near-zero nucleation rate at 0.33 < *f* < 0.95 occurred for the given cooling rate.

The mechanism of the austenite-to-pearlite transformation in steel B under continuous cooling conditions differed from that in steel BX. At the cooling rates of 0.5 K/s and 0.3 K/s, one-dimensional nucleation and growth at a near-zero nucleation rate were not observed, while two- and three-dimensional nucleation and growth at a constant nucleation rate occurred (2 < *n* < 4). At the cooling rates of 0.1 K/s and 0.05 K/s, three-dimensional nucleation and growth at a constant nucleation rate were observed (*n* was about 4). 

For steel D, as for steel B, one-dimensional nucleation and growth at a near-zero nucleation rate was not observed. Two- and three-dimensional nucleation and growth were observed for steel D at all cooling rates used (2 < *n* < 4).

## 4. Discussion

With the exception of Mo, the chemical composition of BX, B and D steels was similar to the composition of the 55CrMo steel studied in [8]. However, for the steels understudy, hypoeutectic ferrite was observed in very small amounts (Figure 2). The influence of ferrite on austenite decomposition kinetics was abandoned due to a very small amount of this phase. The local activation energy for the austenite-to-pearlite transformation *E_f_* of all the steels decreased as the volume fraction of the transformed phase increased (Figure 6). This trend was observed among the amorphous Finement alloy in [11], bainitic steel DST in [12] and carburized steel 25CrNi3Mo in [13], and it followed from the analysis of Equations (1) and (2). It can be concluded from Figure 6 that the alloying of steel 54SiCr6 with copper led to a significant decrease in *E_f_*, while alloying with silicon led to a slight change in the *E_f_* of this steel. 

In [8], the average activation energy for the transformation of austenite into pearlite, $\overset{-}{E_{f}}$, was 726,400 J/mol. To compare the obtained results with the data [8], $\overset{-}{E_{f}}$ was calculated for the steels under study in the same way as in [8]. It turned out to be 602,097 J/mol, 506,590 J/mol and 495,796 J/mol for steels BX, B and D, respectively. Taking into account the chemical composition of the steel studied in [8] and the steels in this study, it can be said that alloying with molybdenum apparently also has an effect on the activation energy for the austenite-to-pearlite transformation.

**Figure 8:**
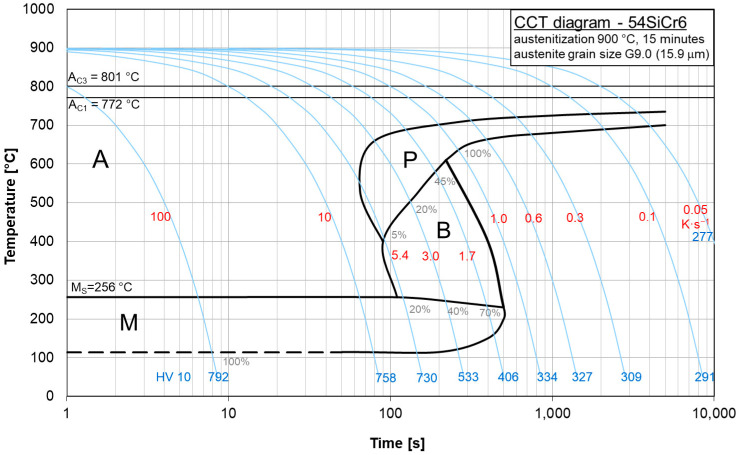
CCT diagram of steel BX.

**Figure 9:**
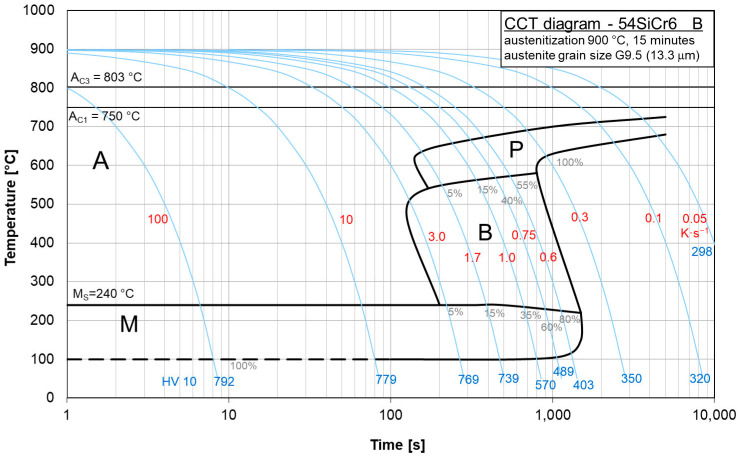
CCT diagram of steel B.

The copper influence was followed in the region of low cooling rates of the CCT diagrams for steels BX (Figure 8) and B (Figure 9). In steel BX (copper content 0.12 wt.%) the austenite-to-pearlite transformation began at higher temperatures and earlier than in steel B (copper content of 1.48 wt.%) for all the cooling rates. Moreover, the duration of this process for steel BX was shorter than for steel B. Evidently, due to a decrease in the local activation energy, the copper alloying led to a shift and expansion of the CCT diagram in the region of low cooling rates. 

These diagrams can be used to represent what types of phase transitions will occur in 54SiCr depending on the copper content upon slow cooling.

The calculated dependences of the local Avrami exponent *n* on the volume fraction of the transformed phase *f* for all the studied steels were qualitatively similar to those reported in [11,12,13], with a strong increase in *n* at *f* < 0.1 and a moderate increase in *n* at *f* > 0.9 and an increase in *n* with a decrease in the cooling rate.

To additionally verify the results, the Avrami exponents were also found according to the approach in [23], where Equation (5) was used:

(5) $${\left(\frac{df}{dt}\right)}_{p}=\frac{0.37n\beta E_{pf}}{RT_{p}^{2}}$$

where ${\left(\frac{df}{dt}\right)}_{p}$, *T_p_* and *E_pf_* are the peak transformation rate, temperature and energy transformation for this peak, respectively.

**Figure 10:**
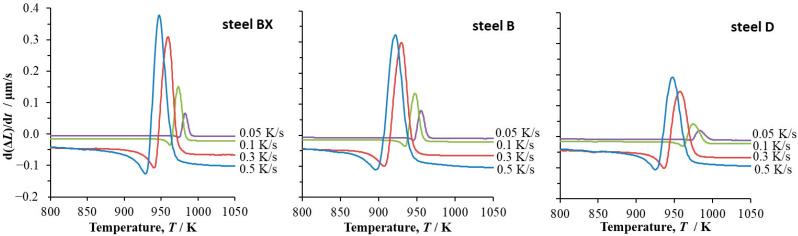
The time derivative of change in length of dilatometric sample, Δ*L*, versus temperature at cooling rates *β* = 0.05 K/s, 0.1 K/s, 0.3 K/s and 0.5 K/s in steel BX, steel B and steel D.

The time derivatives on the dilatometry signal ($\frac{d∆L}{dt}$ − *T*) versus the temperature for steels BX, B and D at all cooling rates used are shown in Figure 10.

Table 3 shows the value of ${\left(\frac{df}{dt}\right)}_{p}$ for steels BX, B and D at cooling rates *β* = 0.05 K/s, 0.1 K/s, 0.3 K/s and 0.5 K/s. The value of *E_pf_* was 537,898 J/mol, 499,664 J/mol and 514,140 J/mol for steels BX, B and D respectively.

For the cooling rates of 0.05 K/s and 0.1 K/s, the values of *n* calculated from Equation (5) turned out to be greater than 4. Therefore, they are not discussed. Such anomalous values of *n* were also found in [24].

The conclusion obtained using the approach in [11,12,13] (Equation (4)) about the one-dimensional nucleation and growth at a near-zero nucleation and at a cooling rate of 0.5 K/s in steel BX was not confirmed using the approach in [23] (Equation (5)). The use of the latter equation for steels B and D allowed for the same conclusion about the mechanism of the austenite-to-pearlite transformation at cooling rates 0.3 K/s and 0.5 K/s as that obtained using Equation (4); the two- and three-dimensional nucleation and growth of the pearlite phase occurred under this cooling regime in steels B and D at a constant nucleation rate. Thus, it can be concluded that for the steels in this study, the use of the approach in [23], in which the data were processed only at the peak transformation rate (Equation (5), Figure 10), provided partial agreement with the results obtained from using the approach in [6,7,8,10,11], which took all the data from the dilatometric measurement into account (Equation (4), Figure 7).

Following [22,23], the local activation energy for the whole austenite-to-pearlite transformation process *E_cf_* was considered as *E_f_*/(*a* + *b*), where *a* is the nucleation exponent and *b* is the growth exponent. In [23], it was estimated that (*a* + *b*) can be replaced by the local Avrami exponent *n*. The average values of the activation energies for the whole austenite-to-pearlite transformation process $\overset{-}{E_{cf}}$ are presented for the investigated steels in Table 4.

Note that similarly to [23], a decrease in the average activation energy for the whole austenite-to-pearlite transformation process was observed as the cooling rate increased.

**Figure 11:**
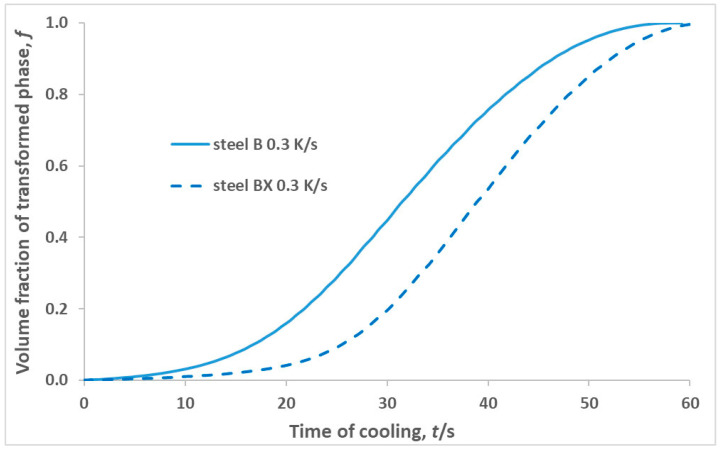
The time dependence of the volume fraction of transformed phase *f* for steels BX and B, subjected to continuous cooling at a rate of 0.3 K/s.

The activation energy of the austenite-to-pearlite transformation was related to the activation energy of the grain boundary diffusion of carbon or carbide forming elements, depending on the chemical composition of the steels under study [25]. Steels BX, B and D and the steel studied in [8] contain a significant quantity of chromium that is prone to the formation of carbides. Therefore, the $\overset{-}{E_{cf}}$ of these steels can be expected to be higher than the activation energy of carbon diffusion through the grain boundary and in the bulk in austenite, which was 40,000–60,000 J/mol in [25], 176,421 J/mol in [26] and 155,397 J/mol in [27], respectively. However, alloying the steel with Cu with more than 0.5 wt% leads to a decrease in the activation energy of carbon diffusion in austenite [28]. This may be the reason that lower values of $\overset{-}{E_{cf}}$ were found for steels B and D than for steel BX and the steel studied in [8]. In addition, this result was consistent with the more intense kinetics in steel B compared to steel BX found in metallographic studies (Figure 2) and the evolution of the volume fraction of the transformed phase with time obtained using a dilatometric experiment for steel BX and B, subjected to continuous cooling at a rate of 0.3 K/s (Figure 11).

It is necessary to discuss the correspondence between the results of the metallographic study and the kinetics analysis of the dilatometric experiment. Three-dimensional nucleation and growth were more likely in bulk, while one-dimensional nucleation and growth were more likely at grain boundaries, in particular on the PAG in the BX, B and D steels studied. The conclusion that the pearlite particles at the PAGs grew faster than inside the PAGs in both steels BX and B at the advanced stage was consistent with the conclusion from the kinetics analysis of dilatometry study about one-dimensional nucleation and growth at a near-zero nucleation rate at *f* > 0.20 in steel BX. However, it did not contradict the conclusion about two-dimensional nucleation and growth in steel B. The explanation of other revealed features of the metallographic study of these steels requires additional investigations.

## 5. Conclusions

The continuous phase transformation of 54SiCr6 steel at slow cooling rates of 0.5 K/s, 0.3 K/s, 0.1 K/s and 0.05 K/s and with different contents of silicon and copper were studied using dilatometry and metallography. The local activation energy for the phase transition *E_f_* and the local Avrami exponent *n* were selected as kinetics parameters. An analysis of the CCT diagrams allowed us to conclude that an increase in the copper content leads to a decrease in temperature and a late onset of the austenite-to-pearlite transformation and an increase in its duration for all the cooling rates used. Evidently, due to a decrease in the local activation energy, the copper alloying led to a shift and expansion of the CCT diagram in the region of low cooling rates.

The dilatometry study and the metallography studies showed the following results for steels BX, B and D:

1. A predominantly pearlite microstructure was formed as a result of cooling at the rates of 0.5 K/s, 0.3 K/s, 0.1 K/s and 0.05 K/s.
2. *E_f_* of all investigated steels decreased as the volume fraction of the transformed phase increased.
3. The alloying of steel 54SiCr6 with copper led to a significant decrease in *E_f_*, while alloying with silicon led to a slight change in *E_f_*.
4. The kinetics of the austenite-to-pearlite transformation process in steel BX was characterised by the following features:
  - at cooling rates of 0.5 K/s, 0.3 K/s and 0.1 K/s, two- and three-dimensional nucleation and growth occurred at a constant nucleation rate and at a volume fraction of transformed phase *f* < 0.20, and one-dimensional nucleation and growth occurred at a near-zero nucleation rate at *f* > 0.20.
  - at a cooling rate of 0.05 K/s, there was two- and three-dimensional nucleation and growth at a constant nucleation rate at 0.14 < *f* < 0.33 and one-dimensional nucleation and growth at a near-zero nucleation rate at 0.33 < *f* < 0.95.
5. The kinetics of the austenite-to-pearlite transformation process in steels B and D was characterised only by two- and three-dimensional nucleation and growth at a constant nucleation rate.

## Figures and Tables

**Table 1:** Chemical compositions of the steels studied, wt.%.

Steel	C	Si	Mn	Cr	Cu	Fe
BX	0.57	1.51	0.68	0.75	0.12	Bal.
B	0.56	1.54	0.70	0.77	1.48	Bal.
D	0.57	2.49	0.75	0.77	1.47	Bal.

**Table 2:** Conditions of individual stages of pearlitic transformation–finish temperature (*T*) and time (*τ*) of slow cooling.

Stage	BX		B		D	
	*τ* (s)	*T* (°C)	*τ* (s)	*T* (°C)	*τ* (s)	*T* (°C)
Initial	687	694	783	665	687	694
Advanced	700	690	797	661	703	689
More advanced	707	688	803	659	710	687
Later	720	684	820	654	730	681

**Table 3:** Derivative ${\left(\frac{df}{dt}\right)}_{p}$, temperature *T_p_* and Avrami exponent *n* at the peak transformation rate for steels BX, B and D cooled at cooling rates *β* = 0.05 K/s, 0.1 K/s, 0.3 K/s and 0.5 K/s.

*β* (K/s)	Steel BX			Steel B			Steel D		
	${\left(\frac{df}{dt}\right)}_{p}$	*T_p_*	*n*	${\left(\frac{df}{dt}\right)}_{p}$	*T_p_*	*n*	${\left(\frac{df}{dt}\right)}_{p}$	*T_p_*	*n*
0.05	0.0729	982.4 K	58.7	0.0609	955.6 K	50.0	0.0435	983.1 K	36.8
0.1	0.0551	973.2 K	21.8	0.0489	974.2 K	19.7	0.0369	974.2 K	15.3
0.3	0.0361	959.7 K	4.6	0.0326	929.8 K	4.2	0.0275	957.4 K	3.7
0.5	0.0279	947.5 K	2.1	0.0244	947.3 K	1.8	0.0236	947.3 K	1.9

**Table 4:** Average of activation energy for the whole austenite-to-pearlite transformation process $\overset{-}{E_{cf}}$ for steels BX, B and D, cooled at cooling rates *β* = 0.05 K/s, 0.1 K/s, 0.3 K/s and 0.5 K/s.

*β* (K/s)	Steel BX	Steel B	Steel D
0.05	651,471 J/mol	193,252 J/mol	180,502 J/mol
0.1	567,569 J/mol	171,601 J/mol	175,070 J/mol
0.3	525,831 J/mol	-	130,535 J/mol
0.5	461,060 J/mol	-	118,921 J/mol

## Data Availability

The data presented in this study are available on request from the corresponding author.
